# Supplementary material for: Using big sequencing data to identify chronic SARS-Coronavirus-2 infections
Source: Nat Commun. 2024 Jan 20;15:648. doi: 10.1038/s41467-024-44803-4 (PMC10799923; doi:10.1038/s41467-024-44803-4)
Supplement: Supplementary file 1 — Supplementary Information [file 41467_2024_44803_MOESM1_ESM.pdf]

Using big sequencing data to identify chronic SARS-Coronavirus-2 infections
Supplementary material

Sheri Harari*, Danielle Miller*, Shay Fleishon, David Burstein & Adi Stern

This supplementary information contains figures, tables and supplementary datasets related to the publication. All Supplementary Datasets described are deposited to the Zenodo database under accession code 10.5281/zenodo.10338988 (https://doi.org/10.5281/zenodo.10338988).

Supplementary Text
Mutational spike S1 differences among groups

Supplementary Tables

Supplementary Table 1 - Variant definition using NextStrain clades.
Supplementary Table 2 - Country distribution across the 271 chronic-like clades divided by continent.
Supplementary Table 3 - Slopes for regression lines of mutations against sampling dates.

Supplementary Figures

Supplementary Figure 1 - Per-variant clade size and time interval distributions
Supplementary Figure 2 - Distribution of sex, age and mutations among all groups
Supplementary Figure 3 - Distribution of Sackin index across 271 chronic-like clades
Supplementary Figure 4 - Global prevalence of RBD convergent mutations across time
Supplementary Figure 5 - Language model Embedding representation and training.
Supplementary Figure 6 - Classification performance assessment
Supplementary Figure 7 - Per-variant clade size and time interval distributions for unknown predictions

Supplementary Datasets

Supplementary Dataset 1 – Chronic-like samples data and identifiers
Supplementary Dataset 2 – Control samples data and identifiers
Supplementary Dataset 3 – USHER down sampled phylogenetic trees and Sackin index measurements
Supplementary Dataset 4 – Examples of chronic like clades presented in Figure 2 and 4
Supplementary Dataset 5 – Regression evolutionary clade rates
Supplementary Dataset 6:

1. Language model corpus
2. Chronic-like clades and controls sentences for classification.
3. Model performance tables
4. LIME explainability results tables
5. Clades with unknown data predictions
6. Fitness of the most explainable mutations

Supplementary Dataset 7 – GISAID identifiers.

Supplementary text

Mutational spike S1 differences among sets of clades

For each set of clades (chronic-like, *bona fide*, control), we calculated the average number of substitutions that occurred in spike S1 subunit (genomic positions 21,563-23,624 according to the Wuhan reference genome). We found on average 1.15, 2.78, and 0.52 substitutions per clade for the chronic-like, *bona fide*, and control sets, respectively. A Mann-Whitney U test was performed on all pairwise comparisons of sets, corrected for multiple comparisons, revealing significant differences in all pairwise combinations. While this supports our observation that chronic-like and *bona fide* are both higher than control clades, we will explain why we believe there is a higher average rate of spike S1 mutations in *bona fide* compared to chronic-like clades. We hypothesize that this is caused by two reasons: (a) Sampling bias within the *bona fide* group. Indeed, one of the publication we relied on [51] specifically targeted chronically infected patients who displayed drug resistance, thus biasing the sample towards more mutated sequences. (b) Moreover, it is likely that our set of chronic-like clades are enriched for chronic infections, but nevertheless may include non-chronically infected individuals.

Supplementary Table 1. Variant definition using NextStrain clades[1,2] .

**Variant**	**NextStrain clade**
Pre VOC	19A,19B,20A,20B,20C,20E,20G,20D
Alpha	20I
Delta	21J,21I
Omicron	21K,21L,22A,22B,22C
Other	20H,20J,21F,21B

Supplementary Table 2. Country distribution across the 271 chronic-like clades divided by continent. Lines in bold represent the country with the largest number of chronic-like clades in a continent.

**Continent**	**Countery**	**Chronic-like clades**
Africa	South Africa	2
Asia	Indonesia	1
	Japan	2
	Israel	5
	India	13
Europe	Croatia	1
	Slovakia	1
	North Macedonia	1
	Austria	3
	Romania	3
	Netherlands	3
	Ireland	3
	Luxembourg	7
	Belgium	8
	Germany	11
	Sweden	14
	Italy	24
	Slovenia	30
	Spain	33
	France	48
North America	Mexico	2
	Canada	35
	United States of America	55
Oceania	Australia	7
South America	Argentina	1
	Peru	1
	Brazil	5

Supplementary Table 3. Slopes for regression lines of mutations against sampling dates per variant. Variants inspected are Alpha (*n*=1,733), Delta (*n*=4,794), BA.1 (*n*=1,946) and BA.2 (*n*=2,822). T-tests described are two-tailed tests, for correction multiple testing the FDR correction was used.

Variant	Label	Overall[1]	Synonymous[2]	Non-synonymous[3]	Spike non-synonymous[4]	S1 non-synonymous[5]
Alpha	control	12.50	6.11	7.62	3.01	3.13
	chronic-like	16.32	6.72	13.02	8.06	8.06
Delta	control	14.67	6.88	8.96	2.17	1.52
	chronic-like	10.38	4.48	7.98	4.88	4.55
BA.1	control	10.65	5.20	6.95	2.63	2.10
	chronic-like	22.51	5.97	18.97	10.25	9.42
BA.2	control	10.90	5.66	6.58	2.90	3.21
	chronic-like	15.55	5.087	13.02	8.69	9.50

* T-test for chronic-like vs. control is significant for all pairwise comparisons ($p<10^{-5}$) except for synonymous ($p=0.5$).

Within variant testing:
1 ANOVA: p =0.05, Tukey: Delta-BA.1 p=0.03, all other pairs are non significant
2 ANOVA: p =0.87, Tukey: all pairs are non significant
3 ANOVA: p =0.019, Tukey: Delta-BA.1 p=0.013, all other pairs are non significant
4 ANOVA: p =0.10, Tukey: all pairs are non significant, Delta-BA.1 borderline p=0.06
5 ANOVA: p =0.09, Tukey: all pairs are non significant, Delta-BA.1 borderline p=0.07
ORF1a and ORF1b ANOVAs are non significant for any group presented.

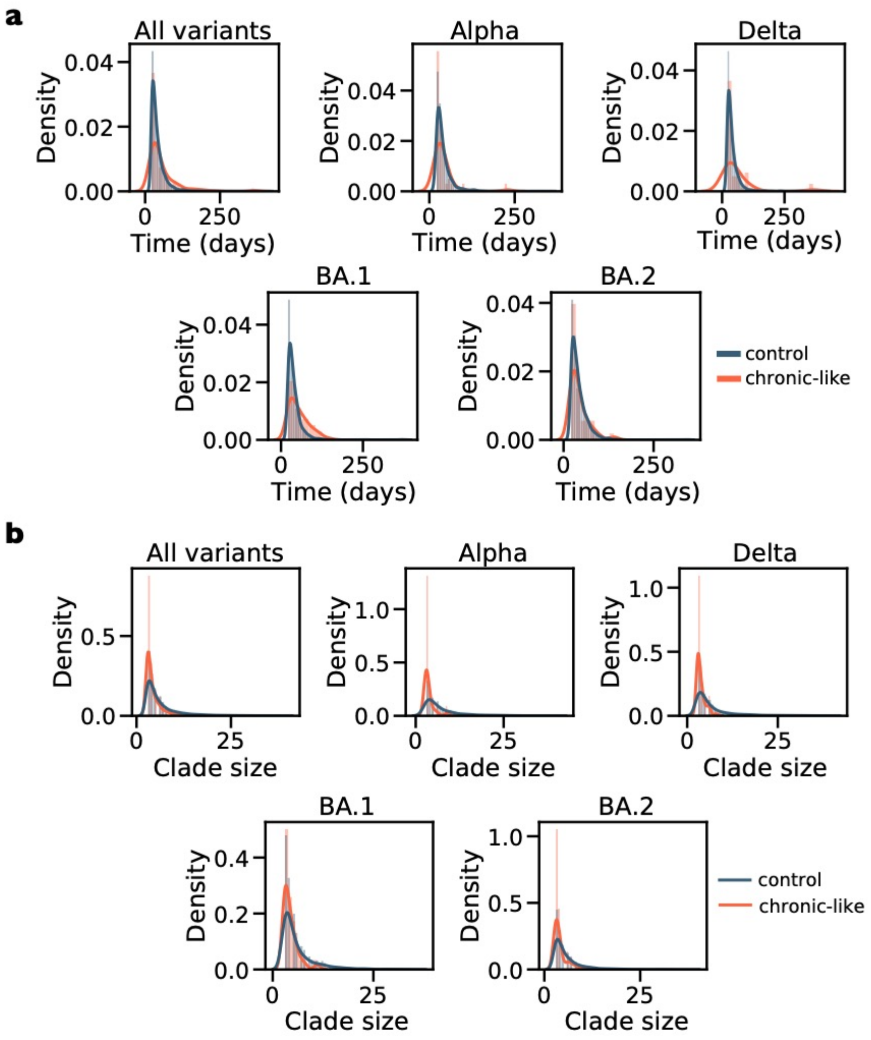

Supplementary Figure 1. Per-variant clade size and time interval distributions separated by class. (a) Time interval distribution in days of control clades (*n*=15,163) and chronic-like clades (*n*=271) (b) Clade size distribution. Each stratified sample of *n*=271 clades from the control, is not significantly different in clade size and time from the relevant chronic-like distribution (Mann Whitney, $p > 0.05$).

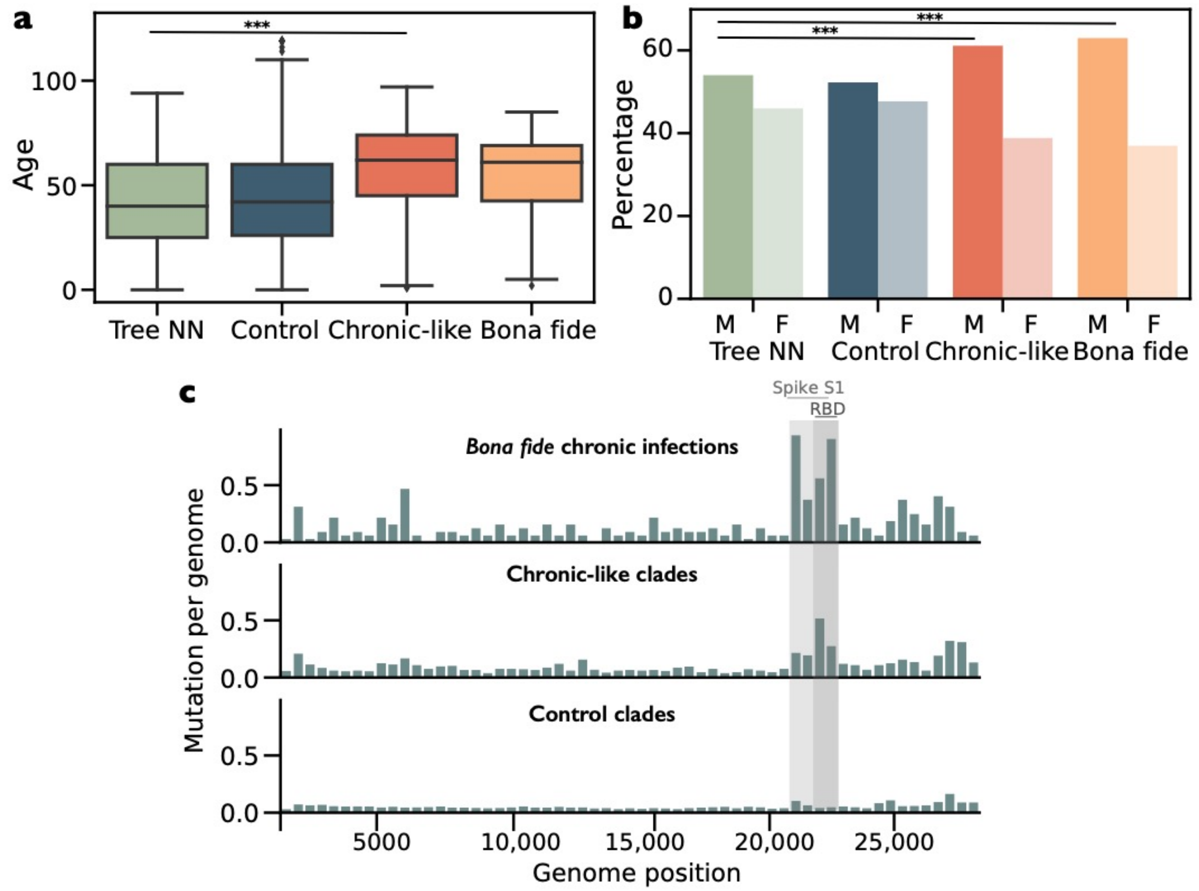

Supplementary Figure 2. Distribution comparison of age and sex. (a) Tree nearest neighbors (NN) age distribution is significantly lower than chronic-like clades $p < 10^{-4}$, ANOVA and Tukey for pairwise comparisons). Tree NN and *bona fide* chronic infections are with a borderline p-value of $p = 0.06$. (b) Percentage of male/female shown for tree NN (*n*=498), control clades (*n*=15,163), chronic-like clades (*n*=271), and *bona fide* chronic infections (*n*=32). Tree NN percentage of males is significantly lower than chronic-like clades and *bona fide* chronic infections ($p < 10^{-4}$, permutation test and bootstrap). (c) Distributions of mutation per bin per genome along the SARS-CoV-2 genome observed across all bona fide chronic infections, chronic-like clades, and control clades. Substitutions are counted in bins of 500 nucleotides. See supplementary text above for a more thorough explanation on differences among categories.

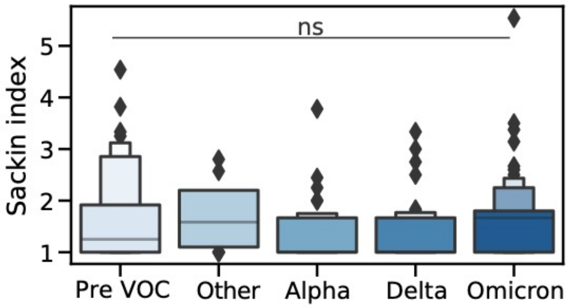

Supplementary Figure 3. Distribution of Sackin index across 271 chronic-like clades, categorized by Nextstrain variant. Variants were classified based on Table S2. An ANOVA test was conducted to assess differences among the variant groups, revealing no statistically significant differences ($p = 0.16$). Counts per variant are described in Figure 1a.

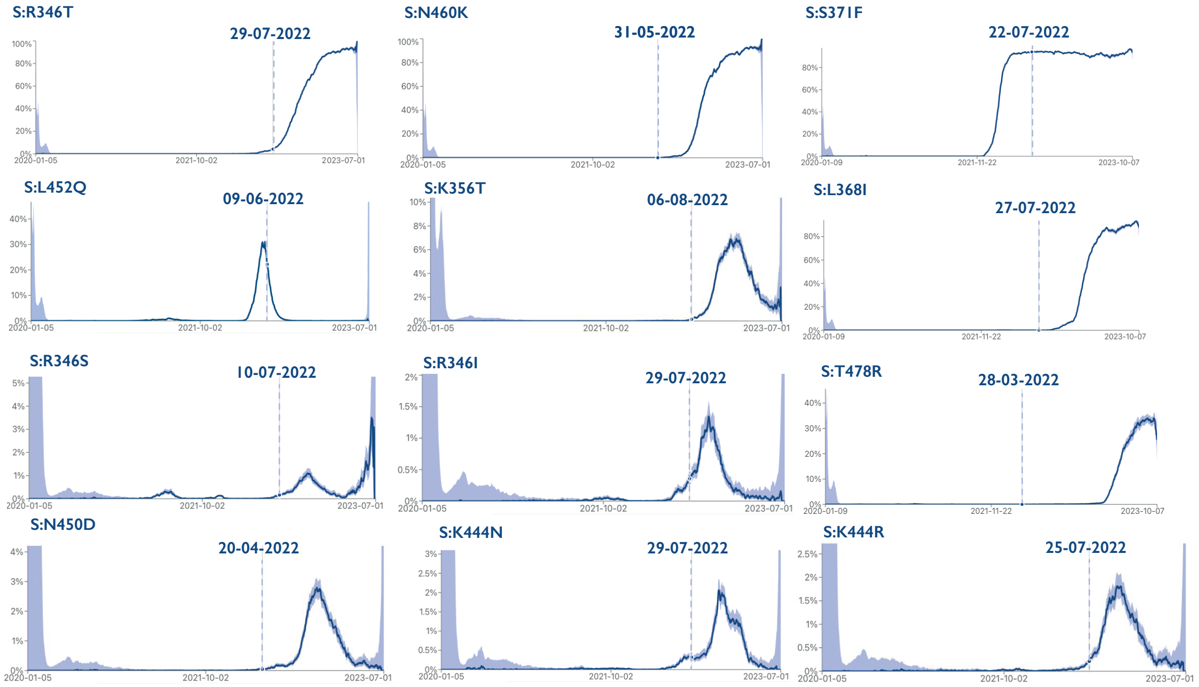

Supplementary Figure 4. Global prevalence of RBD mutations found in BA.1/BA.2 chronic like clades that achieve high global prevalence (Fig. 4c, top nine rows; broken down by mutation type and hence twelve mutations shown). Data were derived from cov-SPECTRUM[3]. Dashed lines correspond to the latest sampling data of a chronic-like clade where the respective mutations were detected**.** Only mutations that exceeded a global prevalence of 1% are shown. For all these mutations, the last date of sampling of the chronic like clades precedes or co-occurs with the rise of the mutations to appreciable frequencies, suggesting predictive values of the chronic-like clades. Interestingly, L452Q reached global prevalence of 30% around May 2022 as part of BA.2.12.1, and S:S371F reached global prevalence of 90% around April 2022, in parallel to some chronic-like clades displaying the respective mutation on a background of a different variant.

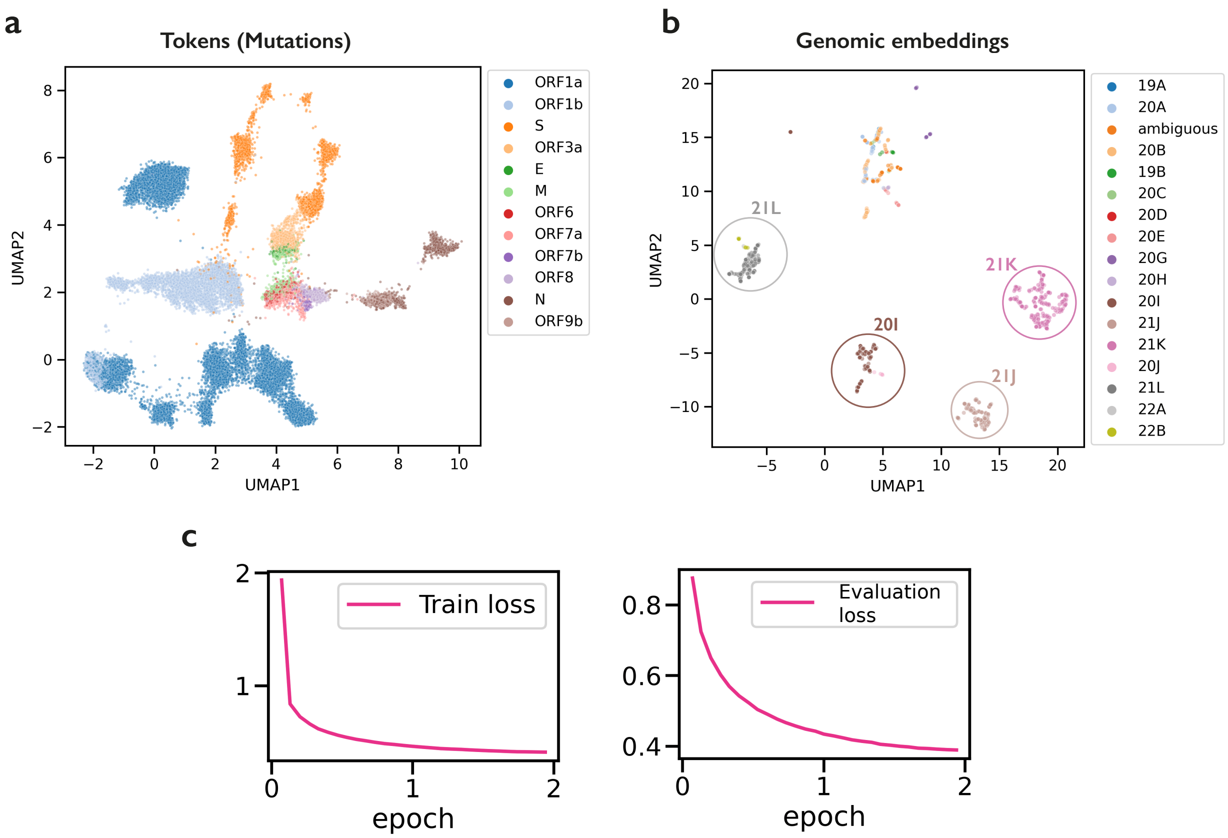

Supplementary Figure 5. Language model embedding representation and training. (a) Token (mutation) embedding projection into 2-dimensional space using UMAP of the trained BERT model. The mutations are color-coded by their genes. This analysis does not take into account the full genome sequence. (b) Genomic embeddings of all sequences of the 271 chronic-like clade (1,337 sequences). The genome embedding was calculated by averaging the embeddings of each token of each sequence. This figure underscores the importance of the genomic embeddings for further biological predictions. (c) Train and Evaluation loss across 2 training epochs.

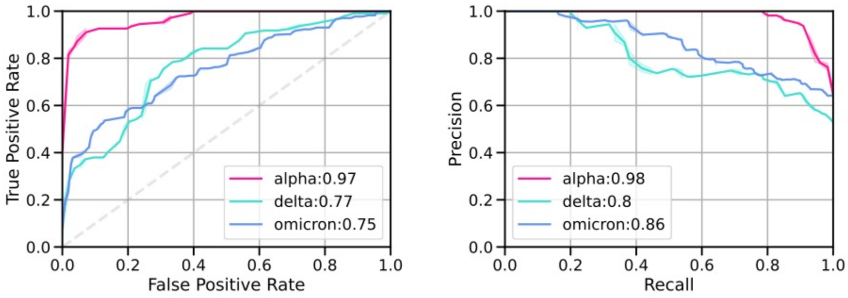

Supplementary Figure 6. Classification performance assessment. ROC and precision recall curves separated by the variant fold in the time and variant cross validation. Values are weighted by clade size and number of mutations in the clade, as larger clads with more mutations are deemed more reliable. The values described in the embedded legend are the area under the curve for both ROC and precision recall graphs. Of note, the classifier performance is reduced from Alpha to Delta and Omicron, likely due to two reasons: (i) The increase in the number of sequences and associated increase in genetic diversity over time, and (ii) The increase in epistatic interactions, particularly notable in the omicron background [4,5].

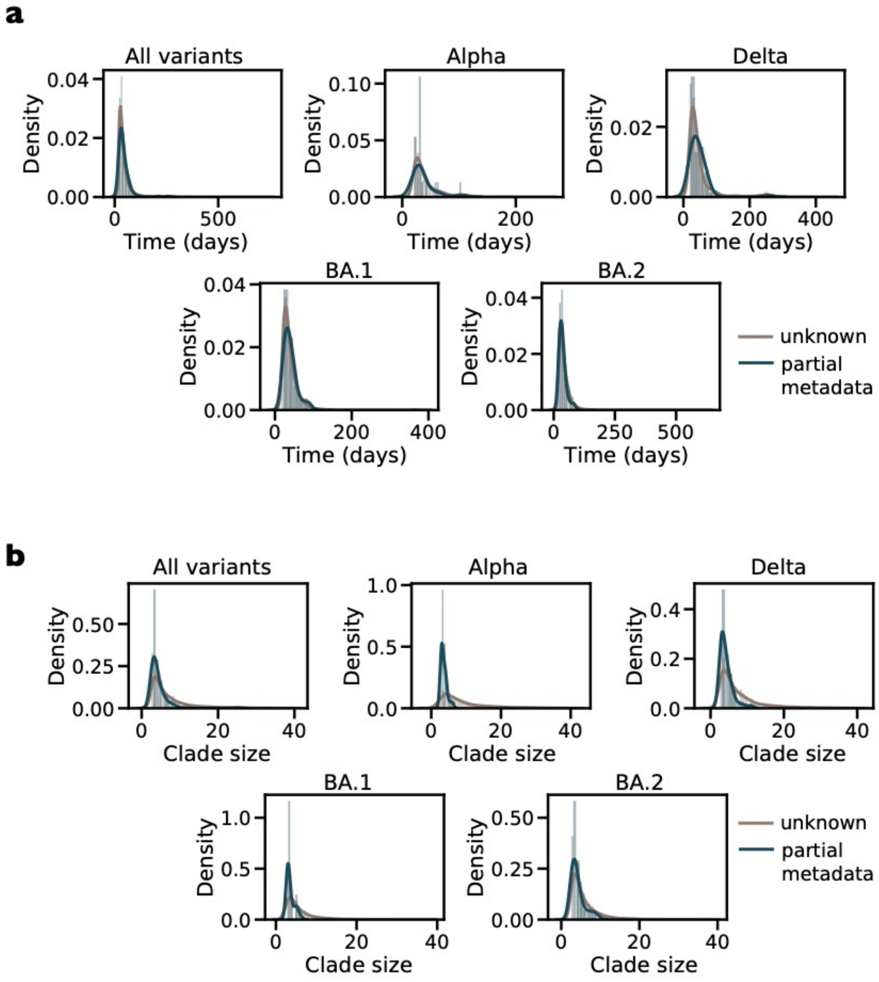

Supplementary Figure 7. Per-variant clade size and time interval distributions for unknown predictions (i.e., predictions with lacking metadata). (a) Time interval distribution in days for unknown group (n= 18,760) and partial metadata (n= 147) (b) Clade size distribution.

References

1. Hadfield, J. *et al.* Nextstrain: real-time tracking of pathogen evolution. *Bioinformatics* **34**, 4121–4123 (2018).
2. Aksamentov, I., Roemer, C., Hodcroft, E. & Neher, R. Nextclade: clade assignment, mutation calling and quality control for viral genomes. *J. Open Source Softw.* **6**, 3773 (2021).
3. Chen, C. *et al.* CoV-Spectrum: analysis of globally shared SARS-CoV-2 data to identify and characterize new variants. *Bioinformatics* **38**, 1735–1737 (2022).
4. Moulana, A. *et al.* Compensatory epistasis maintains ACE2 affinity in SARS-CoV-2 Omicron BA.1. *Nat. Commun.* **13**, 7011 (2022).
5. Starr, T. N. *et al.* Deep mutational scans for ACE2 binding, RBD expression, and antibody escape in the SARS-CoV-2 Omicron BA.1 and BA.2 receptor-binding domains. *PLOS Pathog.* **18**, e1010951 (2022).
