# Supplementary material for: Using big sequencing data to identify chronic SARS-Coronavirus-2 infections
Source: Nat Commun. 2024 Jan 20;15:648. doi: 10.1038/s41467-024-44803-4 (PMC10799923; doi:10.1038/s41467-024-44803-4)
Supplement: Supplementary file 3 — Reporting Summary [file 41467_2024_44803_MOESM3_ESM.pdf]

nature portfolio

Corresponding author(s): Adi Stern

Last updated by author(s): Jul 22, 2023

Reporting Summary

Nature Portfolio wishes to improve the reproducibility of the work that we publish. This form provides structure for consistency and transparency in reporting. For further information on Nature Portfolio policies, see our Editorial Policies and the Editorial Policy Checklist.

Statistics

For all statistical analyses, confirm that the following items are present in the figure legend, table legend, main text, or Methods section.

n/a	Confirmed	
☐	☒	The exact sample size (*n*) for each experimental group/condition, given as a discrete number and unit of measurement
☐	☒	A statement on whether measurements were taken from distinct samples or whether the same sample was measured repeatedly
☐	☒	The statistical test(s) used AND whether they are one- or two-sided<br>*Only common tests should be described solely by name; describe more complex techniques in the Methods section.*
☒	☐	A description of all covariates tested
☐	☒	A description of any assumptions or corrections, such as tests of normality and adjustment for multiple comparisons
☐	☒	A full description of the statistical parameters including central tendency (e.g. means) or other basic estimates (e.g. regression coefficient) AND variation (e.g. standard deviation) or associated estimates of uncertainty (e.g. confidence intervals)
☐	☒	For null hypothesis testing, the test statistic (e.g. *F*, *t*, *r*) with confidence intervals, effect sizes, degrees of freedom and *P* value noted<br>*Give P values as exact values whenever suitable.*
☒	☐	For Bayesian analysis, information on the choice of priors and Markov chain Monte Carlo settings
☒	☐	For hierarchical and complex designs, identification of the appropriate level for tests and full reporting of outcomes
☒	☐	Estimates of effect sizes (e.g. Cohen's *d*, Pearson's *r*), indicating how they were calculated

Our web collection on statistics for biologists contains articles on many of the points above.

Software and code

Policy information about availability of computer code

Data collection	Data was obtained from publicly available resources, elaborated under the "Data" section.
Data analysis	All data analysis was conducted using scripts and models available in the GitHub repository: https://github.com/Stern-Lab/chronic-covid-mlm.<br>The data used in the analysis is accessible through the provided links.<br>The scripts were implemented in Python 3.10, and deep learning models were trained using Hugging Face Transformers version 4.26.1 with PyTorch version 2.0.0+cu117. To explain the models' predictions, LIME explainability was employed with the lime package version 0.2.0.1.<br>For statistical analyses, regression analysis and ANOVA/Tukey tests were performed using the statsmodels package version 0.13.5. The figures were generated using the seaborn package version 0.12.2 and matplotlib version 3.6.3.<br>The analysis also utilized various Python libraries, including numpy version 1.23.5, pandas version 1.4.3, scipy version 1.10.0, scikit-learn version 1.2.0, and dendropy version 3.<br>Full description of packages and versions is available in the repository.<br><br>To facilitate the training of deep learning models, a single NVIDIA RTX A6000 GPU with 48G RAM and 8 CPUs was employed.

For manuscripts utilizing custom algorithms or software that are central to the research but not yet described in published literature, software must be made available to editors and reviewers. We strongly encourage code deposition in a community repository (e.g. GitHub). See the Nature Portfolio guidelines for submitting code & software for further information.

Data

Policy information about availability of data

All manuscripts must include a data availability statement. This statement should provide the following information, where applicable:

- Accession codes, unique identifiers, or web links for publicly available datasets
- A description of any restrictions on data availability
- For clinical datasets or third party data, please ensure that the statement adheres to our policy

Sequences were downloaded from the GISAID database on September 17th, 2022.
Quality control was performed using Nextclade version 2.5, and GISAID metadata was manually reviewed and corrected for data inconsistencies and misplacements. The UShER phylogenetic tree was kindly provided by Angie Hinrichs on August 25th, 2022.All data supporting this study's findings have been deposited in the Zenodo database under accession code 10.5281/zenodo.10338988 (https://doi.org/10.5281/zenodo.10338988). These data include the chronic-like and control clades identifiers, mutations, and sub-trees. They also include language model raw corpus files, trained models, and all predictive mutations for the chronic-like clades groups. The full description is available in the Supplementary information and in the Readme file in the link provided.

Research involving human participants, their data, or biological material

Policy information about studies with human participants or human data. See also policy information about sex, gender (identity/presentation), and sexual orientation and race, ethnicity and racism.

Reporting on sex and gender	Sex information was obtained from publicly available metadata downloaded directly from GISAID. Approximately 25% of the database provided reliable information regarding the sex of individuals. In cases where reliable data was unavailable, the sex was labeled as "unknown." Dataset included both Females and Males.<br><br>We utilized sex information from previous publications that validated chronically infected individuals to enhance our dataset. This additional data allowed us to conduct a comprehensive analysis of sex-based metadata for data curation, both in case and control selection and in subsequent case and control comparisons
Reporting on race, ethnicity, or other socially relevant groupings	N/A
Population characteristics	Age information was obtained from publicly available metadata downloaded directly from GISAID. Approximately 25% of the database provided reliable information regarding the age of individuals. In cases where reliable data was unavailable, the age was labeled as "unknown." As the obtained ages depicts the entire world population we had age ranges of 0-120.
Recruitment	N/A
Ethics oversight	N/A

Note that full information on the approval of the study protocol must also be provided in the manuscript.

Field-specific reporting

Please select the one below that is the best fit for your research. If you are not sure, read the appropriate sections before making your selection.

☐ Life sciences ☐ Behavioural & social sciences ☒ Ecological, evolutionary & environmental sciences

For a reference copy of the document with all sections, see nature.com/documents/nr-reporting-summary-flat.pdf

Ecological, evolutionary & environmental sciences study design

All studies must disclose on these points even when the disclosure is negative.

Study description	In this study, we conducted an extensive analysis of SARS-CoV-2 sequences to investigate chronic infections using phylogeny and deep learning. Leveraging publicly available sequencing data & metadata from GISAID, we identified chronic-like infections based on clustering patterns and temporal span. Chronic-like clades exhibited characteristics akin to validated chronic infections, such as older age, male predominance, enriched spike mutations, and a "ladder-like" tree topology. Furthermore, we found that chronic infections accelerated the viral evolutionary rate. Remarkably, chronic-like clades could predict future mutations in circulating variants. To manage the vast dataset efficiently, we utilized deep learning models, leading to the discovery of additional chronic-like clades even in sequences lacking age/sex information.
Research sample	The entire GISAID dataset was used and sampled to >11.7 million publicly available SARS-CoV-2 sequences with associated metadata from GISAID. The data was sampled to ensure high quality of the sequencing data as described in the Methods..
Sampling strategy	Sampling was performed to create controls: To ensure robust and accurate analysis when comparing cases and controls, a stratified

Sampling strategy	bootstrap method was employed. This technique accounts for differences in sample sizes between the two groups, allowing for fair and representative comparisons. The stratification process was carried out using random generators from the widely-used numpy and pandas Python packages.<br><br>For the classification of controls, subsampling was implemented using the same stratification approach. This method allowed for the creation of balanced and comparable control subsets, ensuring unbiased and reliable results in the classification analysis.
Data collection	Sequences were downloaded by the first two authors from the GISAID database on September 17th, 2022.<br>The UShER phylogenetic tree was kindly provided by Angie Hinrichs on August 25th, 2022.
Timing and spatial scale	The data utilized for this study was downloaded on September 17th, 2022, and encompasses all available sequences on GISAID from the beginning of the COVID-19 pandemic in Dec. 2019 up until that specific date. These were all the data available at the time of the study.
Data exclusions	Sequences underwent strict quality control, using Nextclade v2.5 with adjusted mixed sites threshold (30) and exclusion of the "private mutations" criterion. Sequences with final quality scores of "bad" or "mediocre" were discarded, retaining only those labeled as "good." Ambiguous or conflicting date entries (missing/partial dates or submission dates earlier than collection date) were excluded from the analysis. Additionally, we masked specific positions\ mutations flagged as problematic by the literature.
Reproducibility	N/A. Irrelevant for clinical samples.
Randomization	N/A. Irrelevant for clinical samples.
Blinding	N/A. Irrelevant for clinical samples.

Did the study involve field work? ☐ Yes ☒ No

Reporting for specific materials, systems and methods

We require information from authors about some types of materials, experimental systems and methods used in many studies. Here, indicate whether each material, system or method listed is relevant to your study. If you are not sure if a list item applies to your research, read the appropriate section before selecting a response.

Materials & experimental systems

n/a	Involved in the study
☒	☐ Antibodies
☒	☐ Eukaryotic cell lines
☒	☐ Palaeontology and archaeology
☒	☐ Animals and other organisms
☒	☐ Clinical data
☒	☐ Dual use research of concern
☒	☐ Plants

Methods

n/a	Involved in the study
☒	☐ ChIP-seq
☒	☐ Flow cytometry
☒	☐ MRI-based neuroimaging
